# Context effects on the processing hierarchy of vocal expressions

**DOI:** 10.1093/cercor/bhaf343

**Published:** 2026-01-13

**Authors:** Patricia E G Bestelmeyer, Delyth Evans

**Affiliations:** School of Psychology, Brigantia Building, Bangor University, Bangor, Gwynedd, LL57 2AS, United Kingdom; School of Psychology, Brigantia Building, Bangor University, Bangor, Gwynedd, LL57 2AS, United Kingdom

**Keywords:** carry-over design, context, fmri adaptation, kuleshov effect, vocal expressions

## Abstract

Context is crucial for interpreting emotional expressions. Behavioral work has consistently demonstrated the powerful impact of emotional context on disambiguating affective expressions within and across modalities. A theoretical framework suggests that context affects vocal emotion perception at all stages of the neural processing hierarchy, including primary auditory cortex. Using functional neuroimaging, we explored how emotional context images influence the perception of subsequently presented vocal emotional morphs taken from fear to pleasure continua. Morphs were embedded in a balanced sequence to enable the investigation of repetition suppression effects, while context images were blocked by emotion. Results revealed that emotionally congruent context-morph pairings enhanced activation in bilateral superior temporal gyri, including bilateral primary auditory cortex. In contrast, emotional incongruence activated bilateral inferior frontal gyri, regions typically associated with domain-general conflict resolution. To determine whether the activation in primary auditory cortex reflects feedforward or feedback processing, we analyzed the effects of context on adaptation to the morphs. Adaptation to vocal emotion was not differentially modulated by context type. Our findings suggest that context information is initially processed independently of the auditory signal and integrated after the adaptation stage, with contextual influences on sensory cortex mediated via feedback mechanisms.

## Introduction

The human voice transmits a myriad of social information, including the speaker’s gender, identity, and emotional state. Interpreting these acoustic signals as meaningful messages is challenging, yet essential for facilitating effective communication. Even when speech information from multiple sensory channels is considered, for example, combining vocal and facial cues, the integration and interpretation of these complex messages remains difficult (McGurk and Macdonald 1976). Research indicates that multimodal inputs are also important in emotion perception because expressions are often ambiguous cues that rely heavily on contextual information. For example, sad facial expressions are more likely perceived as sad if presented with vocal prosody in a congruent emotional tone compared to an incongruent emotional tone (de Gelder and Vroomen 2000). In behavioral studies on emotion perception, such facilitatory effects of stimulus pairs that are congruent with the situational context have been consistently found across different domains, modalities and experimental designs (for example, facial expressions and body postures [Aviezer et al. 2008]; facial expressions and background colors [Frühholz et al. 2011]; facial expressions and emotional scenes [Righart and De Gelder 2008]; body postures and language [Havas et al. 2007]; social perception [Levy 1960]; pictorial influences on emotion perception [Marian and Shimamura 2012]; vocal expressions and music [Liuni et al. 2020]). While context manipulations in perception research need not necessarily be emotional in nature, many studies investigating context effects have focused on discrete emotions expressed in auditory–visual stimulus pairs. Emotional contexts offer an effective model for studying perceptual modulation, as they tend to elicit robust and reliable effects. This makes them a practical and powerful tool for probing how contextual information shapes the interpretation of ambiguous input.

Before turning to the literature on voice perception, it is helpful to consider the face perception literature because voices have often been conceptualized as an “auditory face” (Belin et al. 2004), carrying social information in parallel to that conveyed by the face. Given that voice processing is less well studied, insights from the face perception literature provide a valuable framework for understanding how contextual influences might operate in the auditory modality. There is discrepancy between theoretical models of face processing and the empirical evidence that has examined the timeline of the integration of contextual information with facial emotion perception. According to models of face processing, the representation of facial expressions relies on processes in higher-level areas, in particular superior temporal sulcus (STS) and, to some extent, fusiform face area (eg Haxby et al. 2000; Duchaine and Yovel 2015). However, eye-tracking and ERP data have suggested that integration of information from face and context (eg body posture) occurs early in the visual processing hierarchy (Meeren et al. 2005; Aviezer et al. 2008; Aviezer et al. 2011). In particular, Meeren et al. (2005) documented an enhancement of the occipital P1 component 116 ms after the onset of the face-body stimulus pair when the pair was emotionally incongruent. The authors interpret their finding as evidence of a rapid neural mechanism that is sensitive to the degree of congruence between simultaneously presented emotional face-body stimuli and therefore implies early integration of emotional face-body stimuli.

In contrast, Teufel et al. (2019) used a behavioral adaptation paradigm to investigate the question of early versus late integration of context effects on facial expression categorization. The authors replicated the biasing effect of body context on facial expression categorization but also showed that adaptation and the resulting high-level aftereffect sizes are unaffected by simultaneously presented body context. The authors concluded that facial expressions and body context are processed in parallel but separate streams and that the integration of information from these channels occurs further downstream, *after* the level of adaptation. Teufel et al.’s study is therefore in line with well-established face processing models.

Consistent with the idea of a later integration of context are studies using functional MRI (eg Müller et al. 2011). Here effects of context, typically assessed using emotionally congruent versus incongruent stimulus pairs are found in superior temporal cortex, not primary visual areas (eg Jeong et al. 2011). There is a clear discrepancy in the interpretation of findings between the aforementioned ERP studies (Meeren et al. 2005) and MRI studies (Müller et al. 2011). While Meeren et al. (2005) interpret the occipital P1 enhancement as reflecting early integration of face and body information during congruent compared to incongruent stimulus combinations, this finding could instead simply reflect early incongruence detection.

In the voice perception literature, on the other hand, Schirmer and Kotz (2006) proposed a hierarchical model that predicts the neural underpinnings of emotional voice perception, including the integration of context effects. The first stage of this model consists of low-level acoustic analysis in bilateral auditory cortices. These sensory areas then project to superior temporal sulci (STS) and superior temporal gyri (STG) for more complex processing in which emotionally salient information is synthesized into an emotional “Gestalt” or acoustic object. The STS and STG then feed into frontal areas for higher-order cognition (eg evaluative judgments of prosody). In contrast to the face processing models, this model explicitly incorporates effects of context along each of the three stages of the auditory processing hierarchy. The model explicitly posits that context information exerts a feedforward influence on vocal emotion perception, affecting even the early stages of sensory processing. However, this proposition has received limited empirical attention.

Across a range of auditory and visual stimulus types, previous research has examined the integration of context effects using a congruence manipulation between emotional stimulus pairs (eg faces and music). We know that congruent stimulus pairs enhance the emotional experience compared to incongruent pairs (eg faster emotion recognition; de Gelder and Vroomen 2000; Dolan et al. 2001; Pell et al. 2022). Early neuroimaging work has shown that congruent cross-modal stimuli modulate activation in the amygdala and fusiform cortex (Dolan et al. 2001). However, there appears to be consensus in later, well-powered studies that congruence consistently increases activation in the superior temporal gyrus when manipulating the level of congruence of pairs displaying discrete emotions in humans (eg Jeong et al. 2011; Gao et al. 2020, 2023), and in non-human animals like the macaque (eg Froesel et al. 2022). It is less clear which brain areas deal with incongruence detection because these are less consistently reported (eg Ethofer et al. 2006 vs. Watson et al. 2013). However, when incongruence effects are reported, research has often cited the inferior frontal gyri (Gao et al. 2023). Previous research suggests that these areas likely reflect domain-general conflict monitoring rather than integration-specific processes (Nelson et al. 2009; Zaki et al. 2010).

To our knowledge, the question of whether visual context affects auditory sensory areas during emotional sound perception has not been directly explored. Research has shown in various species and through different imaging techniques that visual stimuli can affect primary auditory cortex (eg Ghazanfar et al. 2005; Pekkola et al. 2005; Bizley et al. 2007; Kayser et al. 2007, 2008), and auditory input can affect early visual areas (Vetter et al. 2014). Effects of visual stimuli on auditory cortex could originate subcortically, for example, due to multisensory neurons in the thalamus that project to auditory cortex (Ghazanfar and Schroeder 2006; Cappe et al. 2009). It may be more likely, however, that visual influences on auditory cortex are predominantly cortical. In other words, inputs to auditory cortex may be from high-level association areas (eg STG) or come directly from the visual cortex (King and Walker 2012). It is therefore plausible that visual context affects auditory emotion perception, but at what point in the processing hierarchy these interactions occur is unclear.

Typically, studies have investigated context effects on discrete emotions and presented auditory–visual stimulus pairs simultaneously. Given the aforementioned findings of visual influences on auditory cortex, this manipulation of simultaneous presentation makes it difficult to disentangle the question of whether visual context does, or does not, influence early processing stages as predicted by Schirmer and Kotz (2006). Here, we examined the effects of emotional context images on the vocal emotion processing stages using a specific type of adaptation design, also known as a “continuous carry-over” design (Aguirre 2007). Adaptation paradigms have previously been used to probe hierarchical processing of vocal emotion, with repetition suppression revealing sensitivity to vocal expressions in voice-sensitive regions (Bestelmeyer et al. 2014) and, in the case of emotional speech in voice-sensitive regions and orbitofrontal cortex (Ethofer et al. 2009). We created morphed continua between fearful and pleasant vocalizations (Bestelmeyer et al. 2010, 2014). An unbroken and balanced sequence of morphs was interspersed (ie temporally offset) with context images. The images were blocked by valence. We analyzed direct effects of the morphs on brain activation, and we report carry-over (ie adaptation) effects of the morphs. First, we examined the effects of emotional congruence between the context images and the extreme ends of the morphed continua. We hypothesized the effects of congruence to involve the early auditory processing stages of the model, along with previously reported activations in the STG.

Second, we examined whether context influences adaptation to address the question of whether context is integrated early or further downstream in the processing hierarchy. Adaptation paradigms are widely used to infer the level of representation in the processing hierarchy (eg Xu et al. 2008; Bestelmeyer et al. 2010; Bestelmeyer and Mühl 2022). If context is integrated early, at the sensory level, then adaptation should reflect the integrated percept, and effects of context should modulate adaptation. Conversely, if adaptation is unaffected by context, this suggests that integration occurs downstream of the sites of adaptation, which are typically associated with high-level representations rather than low-level sensory features. To test this hypothesis, we calculated the carry-over effects between consecutive stimuli. Here we computed the physical differences between morphs in the stimulus sequence and examined a main effect of context on adaptation. If context effects are integrated early, we expected a main effect of context on adaptation in primary auditory cortex. If context effects are integrated late, at higher levels of cognition, there should be no effect of context on adaptation.

## Methods

### Participants

The experiment consisted of two sessions. In the first session, we screened 42 volunteers from our student cohort for their suitability to take part in the MRI experiment. Of these participants, based on MRI safety and scores in the normal range on the Hospital Anxiety and Depression Scale (Zigmond and Snaith 1983), 34 individuals met all criteria and responded to the invitation to the MRI session. Three of these participants had to be excluded due to excessive movement (> 3 mm) and two due to poor task performance in the scanner (no responses or random responses). The reported analyses are based on 29 participants (18 women; mean age = 22.33 years; SD = 4.01; range = 18 to 34). All participants were reimbursed with course credit for their time. Informed consent was obtained from all individuals, and the study protocol was approved by the ethics committee at Bangor University.

## Materials

### Context images and their validation

**Fig. 1:**
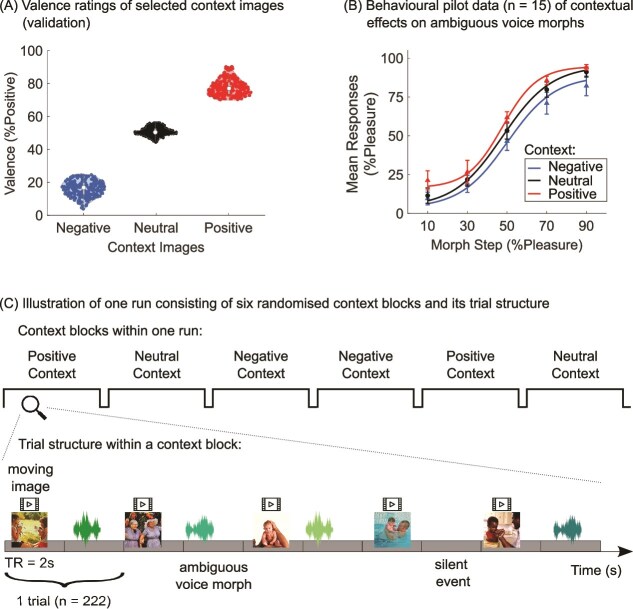
Illustration of image validation data, pilot data and the experimental fMRI paradigm. (A) Mean valence ratings and their distribution for the context images. (B) Behavioral pilot data (*n* = 15) illustrating the biasing effect of context on morphed vocal expressions from fear to pleasure. Error bars represent standard error of the mean (SEM). (C) Schematic illustration of one experimental run of the fMRI paradigm. The top panel shows the six randomized context blocks (two per emotion) within a run. The bottom panel provides a magnified view of the trial structure within a context block, including presentations of a moving image followed by an ambiguous voice morph (or a silent event).

For the initial validation of the image valence, we collected 884 images of negative, neutral, and positive content from a variety of sources (The International Affective Picture System; Lang et al. 2008; and web databases). Pictures were cropped to 400 × 400 pixels in size with a resolution of 300 pixels per inch. Images were given a dynamic movie effect by zooming into the center of the image to enhance vividness (Mobbs et al. 2006). We recruited 12 and 11 participants, respectively, to rate each half of the image set (two randomized sets of 442 images). Participants saw the moving image appear on the screen for 1.4 s. Then an analogue slider was presented, and participants were asked to move it to reflect the perceived valence of the image with extremely negative on the left (value of 0), extremely positive on the right (value of 100) and neutral in the center of the slider (value of 50). The slider was labeled to reflect these instructions. The task took approximately 50 min to complete. We normalized the data using the min-max transform to account for individual differences in the usage of the extremes of the scale. We then selected 222 of the most positive, 222 of the most negative images, and 222 images that received a valence rating closest to 50. The ratings of the contextual images are illustrated in Fig. 1A.

### Voice morphs

Recordings were taken from the Montreal Affective Voices (Belin et al. 2008) in which actors were instructed to produce emotional interjections using the vowel/a/. We used expressions of fear and pleasure because these represent opposite ends of the emotional valence spectrum and can be morphed into continua that are categorically perceived (as illustrated in our pilot data; Fig. 1B). Importantly, these emotions can be morphed in a way that preserves naturalistic acoustic properties. In contrast, alternatives such as happiness (laughter) and sadness (crying) involve rhythmic respiratory-laryngeal bursts (eg laugh syllables or sobs) that would have to be precisely aligned in timing and number to produce naturally sounding voice morphs. This alignment is difficult to achieve with natural recordings and difficult to stage convincingly for actors. We selected expressions of fear and pleasure from four identities (two female) to avoid identity-specific effects of emotion expression. The chosen identities had the highest recognition rates for both expressions. For each identity, we created a continuum consisting of five steps that corresponded to 10/90%, 30/70%, 50/50%, 70/30%, and 90/10% fear/pleasure morphed expressions. We therefore had four continua and a total of 20 morphs which were created using Tandem-STRAIGHT (Kawahara et al. 2008) in Matlab R2014b (The Mathworks, Inc).

Tandem-STRAIGHT performs an instantaneous pitch-adaptive spectral smoothing of each stimulus for separation of contributions to the voice signal arising from the glottal source (including f0) versus supralaryngeal filtering (distribution of spectral peaks, including the first formant frequency, F1). Voice stimuli were decomposed by Tandem-STRAIGHT into five parameters: aperiodicity, duration, frequency, fundamental frequency (f0; the perceived pitch of the voice), and spectrotemporal density. Each parameter is manipulated independently. For each vocal emotion, we manually identified one time landmark with three frequency landmarks (corresponding to the first three formants) at the onset of phonation, and the same number of landmarks at the offset of phonation. Morphed stimuli were then generated by re-synthesis based on the interpolation (linear for time; logarithmic for F0, frequency, and amplitude) of these time-frequency landmark templates (see Kawahara et al. 2008; Schweinberger et al. 2014; for a discussion of the voice morphing technique). All stimuli were normalized for energy (root mean square) before and after morphing.

### Image acquisition and experimental paradigm

All scans were acquired with a Philips 3 Tesla Achieva MR scanner using a 32-channel SENSE head coil. The scanning session consisted of three experimental runs, a voice localizer, and an anatomical scan. For the experimental runs, we used T2*-weighted functional scans (TR = 2 s, TE = 30 ms), with interleaved ascending sequence of 35 slices, no slice gap. Field of view was 240 x 240 x 105 mm, with a voxel size of 3 mm^3^, an acquisition matrix of 80 x 78, a flip angle of 77°, and 510 volumes per run. We acquired three of these functional runs, with each one lasting 17:00 min.

While the emotional context images were blocked, we employed a continuous carry-over design (CCO; Aguirre 2007) for the vocal morphs to measure the effects of one morph upon the next using a first-order serially balanced sequence of stimuli known as type-1-index-1 (Nonyane and Theobald 2007). In this sequence, each stimulus is preceded and followed by itself and every other stimulus an equal number of times and was defined by six stimulus types (five morph steps plus one null event [silence]) totaling 37 stimuli. With every new presentation of a full CCO sequence, we randomized the assignment of stimulus type to the numbers 1 to 6 in the CCO sequence (ie the silent event was stimulus 6 during the first full sequence but then was assigned to stimulus 5 during the second presentation of the full sequence, and so on). Each trial lasted two TRs (4 s) and each full CCO sequence of stimuli was divided by ten TRs of silences (20s). Participants were asked to perform a two-alternative forced choice task (2AFC), in which each morph had to be categorized as either expressing fear or pleasure using an MRI-compatible response box (fORP; Current Designs, Inc.). We asked participants to respond as quickly as possible (see Fig. 1C for illustration of the paradigm). We conducted a behavioral pilot study with 15 volunteers (8 female; mean age = 20.2) to preliminarily validate the presence of a context effect in a 2AFC categorization of the morphs. Results are illustrated in Fig. 1B.

Following the experimental runs, participants completed a “voice localizer” scan (Belin et al. 2000; Pernet et al. 2015). Imaging parameters were the same as for the experimental scans, with the exception that we acquired 310 volumes for the localizer (10:34 min). During this block design, participants passively listened to various sounds. Stimuli were presented in 60 blocks (each lasting 5 TRs), consisting of 20 vocal sound blocks (eg words, vocal expressions, humming), 20 environmental sound blocks (eg objects, instruments, animal sounds), and 20 silent blocks. This localizer allows identification of the temporal voice areas (TVAs) using the vocal versus non-vocal contrast. We asked participants to keep their eyes closed during this functional run. We also acquired a whole brain anatomical scan. This T1-weighted scan had the following parameters: FOV: 240 x 240 x 175, voxel size: 1 mm^3^; 175 slices, with an acquisition matrix of 240 x 224, TR = 12 s, TE = 3.5 ms and a scan duration of 5:38 min.

Auditory stimuli were presented binaurally using the electrostatic NNL headphone system with passive noise attenuation of 30 dB at 1 kHz and enhanced hearing protection (NordicNeuroLab, Inc.). Sounds were presented at an intensity of 80 dB SPL(C) while EPIs were acquired. Visual stimuli were presented on a Cambridge Research Systems BOLDscreen 24 LCD for fMRI, located behind the scanner bore and were viewed via a mirror secured to the head coil. All tasks were implemented in Psychtoolbox-3 (Brainard 1997; Kleiner et al. 2007) for Matlab.

### Data analysis

#### Behavioral data

**Fig. 2:**
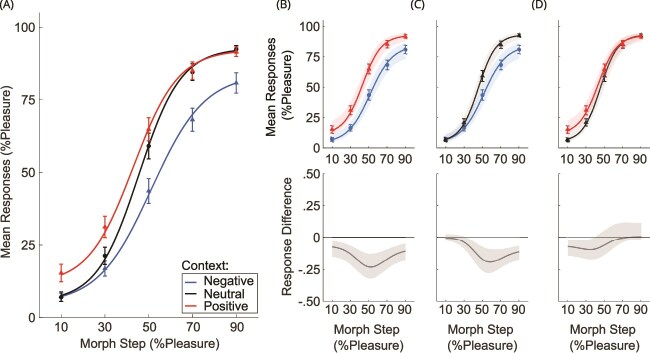
Illustration of the behavioral data acquired during the MRI session. (A) Average psychophysical functions of the behavioral data for the three context conditions. Error bars represent SEM. (B)—(D) Comparison between two context conditions and shaded areas illustrate the bootstrapped CI95% of the fitted curve to participants’ mean responses. Below each curve is the mean difference and CI95% (shaded area) of the differences between two context conditions. Significant differences between conditions typically occurred along the most ambiguous part of the continuum (ie where the CI95% area does not overlap with the y = 0 line) for emotional context conditions compared to neutral (C and D).

We used bootstrapping to assess statistical significance of differences between context conditions (negative, neutral, positive) using Matlab 2023b with the Statistics Toolbox. Each bootstrap sample was derived by randomly sampling participants with replacement. Thus, a participant and their associated data could be selected more than once or not at all, in line with conventional bootstrap methodology (Wilcox 2012). Data from all contexts were included for each sampled participant due to the within-subjects design of the experiment. For each participant, data were averaged as a function of the five morph steps and a psychophysical curve (based on the hyperbolic tangent function) was fitted for each context type (Fig. 2A). We then computed the differences between context conditions. This process was repeated 9999 times, resulting in a distribution of 10,000 bootstrapped estimates of the fit to the psychophysical curves as well as differences between curves for the two conditions. Finally, we calculated the 95% confidence intervals (CI95%) for the fitted curves and the differences between the three context conditions (Fig. 2B to D). A difference between two context conditions was considered significant if the mean difference and its CI95% excluded zero (Cumming 2012).

#### Pre-processing pipeline for the imaging analyses

MRI data were analyzed using SPM12 (The Wellcome Trust Center for Human Neuroimaging, University College London; available at https://www.fil.ion.ucl.ac.uk/spm/software/spm12/) in Matlab 2023b. Pre-processing of the data included AC-PC alignment of the anatomical images, with the same orientation applied to all functional images acquired in the session. Functional scans were corrected for head motion (trilinear interpolation) by aligning all scans to the first scan of the last run, and a mean image was created. The anatomical scan was co-registered to the mean image. Functional and anatomical data were transformed to Montreal Neurological Institute space after segmentation of the anatomical scan. Normalized data were spatially smoothed by applying a Gaussian kernel of 8 mm full width at half maximum.

#### Direct effects of morph step in different contexts (congruence effects)

This analysis tested the first hypothesis that focused on whether emotional congruence between context type and vocal emotion (extreme ends) involves early processing stages (A1). We included the onset times for 15 regressors of interest (5 morph steps x 3 contexts), within block silences, context images, and six movement regressors in the first-level analyses. We computed one contrast for each extreme vocal emotion: one for the extreme ends of pleasure (70/30% and 90/10%) and another for the extreme ends of fear (10/90% and 30/70%). This was done for each of the three context conditions, resulting in a total of six contrasts at the first level of analysis. To examine the main effects of vocal emotion and context, and their interaction (vocal emotion x context), we computed a flexible factorial analysis at the second level. We specified three factors: subject, vocal emotion, and context.

Unless otherwise stated, we report and illustrate activations in text at a threshold of *P* < 0.001 with family-wise error (FWE) correction at the cluster-level (height threshold T = 3.15, ET > 75) from whole brain analyses. For the a priori region of interest (ROI) analysis of activation in primary auditory cortex we made a structural mask of bilateral Brodmann Areas 41 and 42 using the WFU Pickatlas (http://fmri.wfubmc.edu/software/pickatlas). Parameter estimates were extracted in a sphere around the peak voxels of clusters (radius = 3 mm). Statistical significance for the ROI analysis was set at a threshold of *P* < 0.001 with FWE-correction of *P* < 0.05 at the cluster-level.

#### Parametric modulation of physical difference in various contexts (carry-over effect)

This analysis tested the second hypothesis that focused on whether there is an effect of context on adaptation to voice emotion. The analyses of the carry-over effect (ie adaptation or repetition suppression) followed Aguirre (2007) and employed a parametric modulation analysis (Büchel et al. 1998) at the first level. The design matrix included, separately for each context condition, the voice onset events of *n* + 1 trials as the first regressor, and two parametric modulators: the morph step, followed by the physical difference between a morphed stimulus n and the one immediately following it (*n* + 1). The physical difference was calculated as the absolute difference in morph step between n and *n* + 1. For example, if the 50/50% pleasure/fear morph was followed by a 70/30% pleasure/fear morph, the absolute physical difference was 20%. The design matrix also included the onsets of all first sounds of each CCO sequence, silent null events within blocks (excluded from physical difference calculations), the onsets of the contextual images and the six movement regressors. Onsets of first sounds in each sequence were modeled as a separate regressor because no sound preceded it and therefore no carry-over effect could be computed (see Bestelmeyer et al. 2014; Bestelmeyer and Mühl 2022). We report activations in text at a threshold of *P* < 0.001 with FWE-correction at the cluster-level (height threshold T = 3.41, ET > 75).

To determine whether context modulated the physical difference regressor, we conducted a one-way ANOVA (within-subject). This ANOVA included three contrast images (one per context) for the parametric modulator. Unless otherwise stated we report and illustrate activations at a threshold of *P* < 0.001 with FWE-correction at the cluster-level (height threshold F = 7.83) for whole brain analyses.

## Results

### Behavioral data

Figure 2A illustrates the fitted functions for each context condition, along with error bars (standard error of the mean; SEM) for the behavioral data obtained during the scanning session. Figure 2B to D show the fitted functions for two context conditions, error bars (SEM) and the CI95% intervals. The CI95% of the differences between two context conditions are shown below each fitted function and indicate a significant context effect for each comparison when not including zero. The most pronounced difference was observed between the positive and negative context conditions across all morph steps (Fig. 2B). In general, context effects were largest around the 50% morph, the mathematically and perceptually most ambiguous point on the continuum.

### Imaging data

#### Direct effect of morph step in different contexts (congruence effect)

Using a flexible factorial, we found a significant main effect of vocal emotion, with increased activation to more fearful morphs (10/90% and 30/70%) compared to morphs expressing more pleasure (70/30% and 90/10%), in the right superior temporal gyrus (peak maximum: 51–4 -7; k = 98; T(140) = 5.31, *P* = 0.05). We found no main effect of context.

**Fig. 3:**
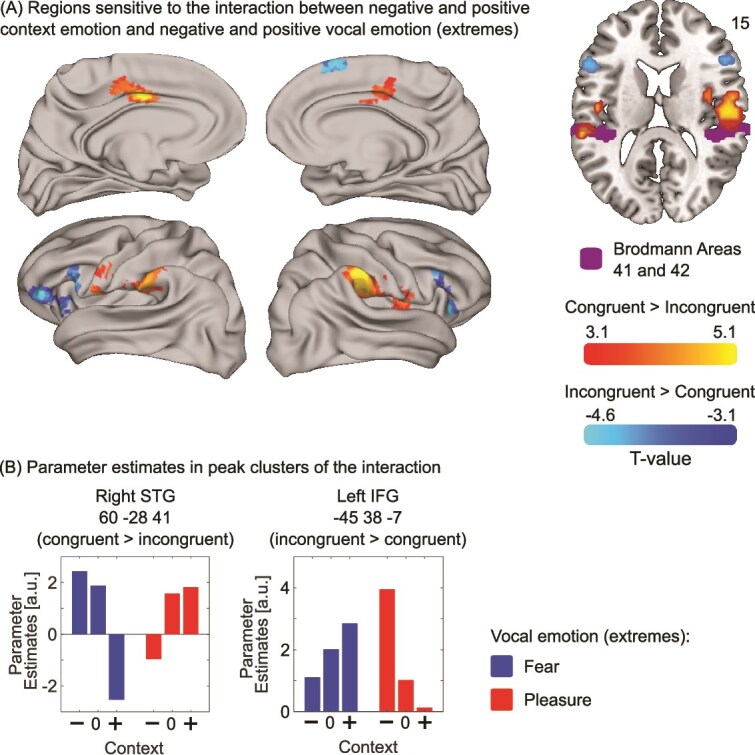
Activation maps of the interactions between context emotion and vocal emotion. (A) Illustration of the activations in response to the interactions between negative (−) and positive (+) context emotion and negative (blue) and positive (red) vocal emotion on an inflated brain template. These interactions correspond to congruent vs incongruent activations in bilateral STG, and for the reverse contrast in bilateral IFG. Activation maps of significant clusters for these interactions are also illustrated on a T1-weighted average structural template and shows the overlap between the activation for the congruent vs incongruent contrast and auditory cortex (Brodmann areas 41 and 42; purple). (B) Illustration of parameter estimates in each peak cluster (left IFG and right STG).

We found significant interactions between vocal emotion and context indicating effects of congruence and incongruence (Fig. 3). Specifically, congruent pairings (ie pleasure morphs in positive context and fearful morphs in negative context) elicited greater activation than incongruent pairings (ie fearful morphs in positive context and pleasure morphs in negative context). This effect was observed in bilateral supramarginal gyrus (right: 60–28 41, k = 464, T[140] = 5.41, *P* < 0.001 and left: −60 -31 29, k = 152, T[140] = 4.29, *P* = 0.01) and left mid-cingulate gyrus (−9 5 35, k = 255, T[140] = 5.07, *P* = 0.001). An a priori ROI analysis revealed significant activation in the left primary auditory cortex (−60–31 20, k = 12, T[140] = 4.03, *P* = 0.03) and borderline significant effect in the right primary auditory cortex (54–13 11, k = 5, T[140] = 3.51, *P* = 0.053).

There was also an effect of the inverse interaction, ie activation to morphs presented in incongruent compared to congruent contexts, in bilateral inferior frontal gyri (left: −45 38–7; k = 237; T[140] = 4.61, *P* = 0.002; right: 51 26–7; k = 116; T[140] = 4.03, *P* = 0.03) and right medial superior frontal gyrus (3 44 41; k = 103; T[140] = 4.23, *P* = 0.04).

#### Parametric modulation analysis of the physical difference in different contexts (carry-over effect)

**Fig. 4:**
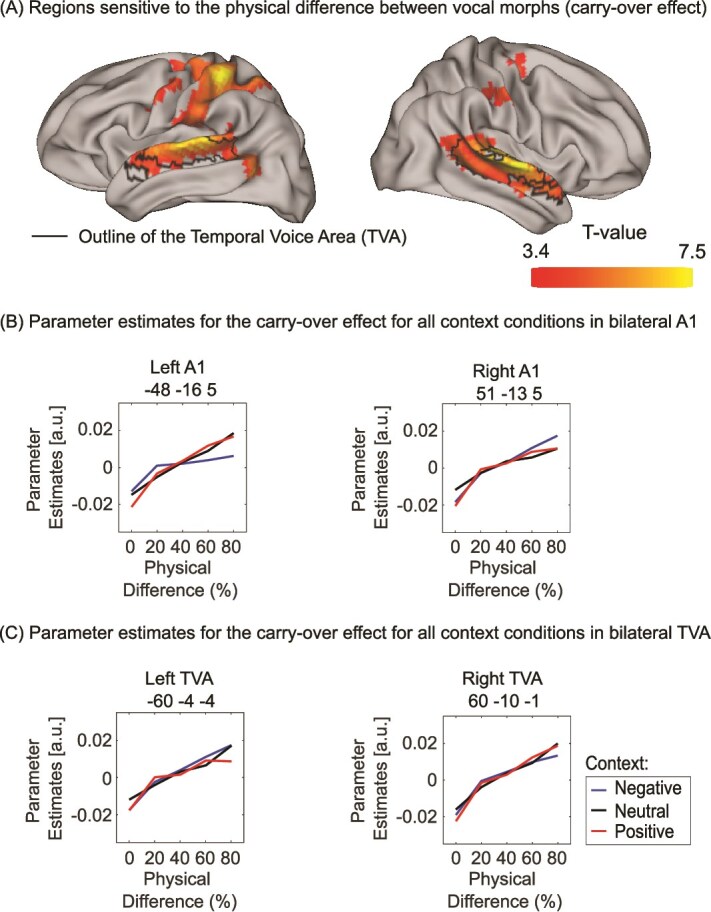
Activation maps for the carry-over effect. (A) Activation maps of the significant correlation between physical difference between consecutive stimuli and BOLD signal (carry-over effect) illustrated on an inflated brain template. Positive correlations (ie increased neuronal adaptation for more similar consecutive stimuli) are evident in areas that largely overlap with bilateral temporal voice areas (black outline). (B) Illustration of parameter estimates from peak maxima within A1 illustrating the correlation between physical difference and BOLD signal independently of context. (C) Illustration of parameter estimates from peak clusters within the TVA illustrating the correlation between physical difference and BOLD signal independently of context.

The parametric modulation analysis of the physical difference regressors revealed significant positive correlations between physical difference and BOLD signal in large clusters covering bilateral superior/midtemporal gyri (right: 60–7 -1, k = 1067, T[28] = 9.06, *P* < 0.001; left with peak cluster in precentral gyrus: −30 -10 56, k = 1991, T[28] = 8.81, *P* < 0.001), right putamen (15 11–10, k = 80, T[28] = 5.70, *P* = 0.037), left mid cingulum (−6–10 56, k = 310, T[28] = 6.59, *P* < 0.001), left precentral gyrus (−57 8 26, k = 91, T[28] = 4.81, *P* = 0.024) and right post-central gyrus (60–13 41, k = 126, T[28] = 4.65, *P* < 0.001). In these regions, activity was greater in response to stimuli that were more acoustically different from the preceding stimulus (larger morph step difference) compared to stimuli with smaller acoustic differences (see Fig. 4A).

First-level contrasts of the three physical difference modulators, separately for each context, were taken to a one-way ANOVA (within-subject). There was no significant main effect of context on the carry-over effect for the whole brain analysis or ROI analyses of A1 and TVA. For detailed illustration of the null effect in our regions of interest, we overlayed the left and right A1 mask and extracted the betas from the peak maximum within the masks from the parametric modulation analysis that included the three physical difference regressors, one per context condition (Fig. 4B). Similarly, we illustrate the parameter estimates for the peak maximum within the left and right TVA (Fig. 4C).

## Discussion

The main objective of this study was to determine whether, and at what stage of the auditory processing hierarchy, situational context influences the perception of vocal emotion. While emotional expressions are often ambiguous and dependent on contextual cues, experimental approaches in neuroscience have frequently overlooked the role of context, treating emotions as isolated, modality-specific phenomena. We demonstrate that context effects were strongest for the perceptually most ambiguous morphs. Schirmer and Kotz (2006) propose a model in which contextual information influences the entire auditory processing hierarchy, from low-level sensory regions (primary auditory cortex) to high-level areas involved in semantic categorization, such as bilateral inferior frontal gyri. Here we examined how emotional context, conveyed through images, modulates the perception of subsequently presented emotionally ambiguous vocal morphs along fear-to-pleasure continua. We show strong behavioral biasing effects in the categorization of ambiguous vocal morphs driven by contextual information. At the neural level, we report strong effects of affective congruence between context images and vocal morphs in bilateral supramarginal gyri, Rolandic opercula and STG, including primary auditory cortex. However, an analysis of the adaptation effect demonstrated no effect of context on adaptation, suggesting that visual and auditory information are initially processed independently and only integrated at later stages, downstream of the sites of adaptation. These findings support the idea that contextual modulation of primary auditory areas is likely driven by feedback mechanisms, rather than the feedforward processes proposed by Schirmer and Kotz (2006). Integration of these cross-modal affective inputs in later stages then supports the emergence of a higher-level emotional representation that is consciously experienced and categorized.

Our behavioral findings are consistent with previous research showing that emotional context affects emotional expression categorization (Bryce et al. 2024). In our study, this biasing effect was most pronounced for the categorization of ambiguous morphs when comparing valenced (positive or negative) to neutral contexts, and extended across the full continuum, including the extreme morphs, when comparing positive and negative contexts. Although it has been unclear where in the processing hierarchy contextual and emotional information are integrated, prior literature typically suggests that these biases are perceptual in nature, rather than arising from higher-order cognitive processes (eg Meeren et al. 2005; Aviezer et al. 2008, 2011). Schirmer and Kotz’s (2006) model on emotional prosody also posits that contextual information influences the entire hierarchy of auditory emotion perception, from the early sensory areas onwards.

Using the “congruency approach” to examine how context modulates vocal emotion perception at the neural level, we found increased activation in response to emotionally congruent events—such as fearful voices following negative context or pleasurable voices following positive context—in clusters spanning the bilateral supramarginal gyrus, Rolandic opercula, and superior temporal regions. This activation was particularly strong in the right hemisphere. Taking a closer look at primary sensory areas using a region of interest analysis, we found that context modulated activation in bilateral primary auditory cortex. These contextual effects on vocal emotion perception in primary sensory areas is consistent with Schirmer and Kotz’s hierarchical model of auditory emotion perception.

Few studies have investigated the effects of affective congruence and often only when examining multisensory integration. Our findings align with recent neuroimaging work using a variety of affective audio-visual stimuli that have identified similar regions (eg music and faces by Jeong et al. 2011; see also Gao et al. 2020, 2023 for a metanalysis). The STG is thought to contain both unisensory and multisensory neurons and may play an important role in cross-modal interaction and integration (Calvert 2001; Beauchamp et al. 2004; Beauchamp et al. 2008). Importantly, the STG appears to respond not only to multisensory affective integration, but also to general content congruency across modalities (eg written and spoken letters [van Atteveldt et al. 2010]; lip movements and speech [Fairhall and Macaluso 2009]. This suggests that the STG may serve as a general hub for integrating congruent information across sensory domains.

Similarly, few studies have focused on the impact of affective incongruence on behavior and BOLD signal. Behaviorally, affective incongruence is characterized by reduced categorization accuracy and slower response times compared to affectively congruent conditions (eg de Gelder and Vroomen 2000; Dolan et al. 2001; Collignon et al. 2008; Wittfoth et al. 2010). At the neural level, a recent meta-analysis by Gao et al. (2023) reported that incongruent compared to congruent stimulus pairs elicit activation in middle and superior frontal gyri, and right inferior frontal as well as middle and superior temporal gyrus (see also Davies-Thompson et al. 2019). Despite design and analytical differences, our findings are in line with this literature in showing increased activation in prefrontal regions during affective conflict. Our results also speak to the broader role of the IFG and superior frontal cortex in domain general conflict resolution and cognitive control processes (Gao et al. 2023). The absence of superior and middle STG activation in our incongruence contrast may reflect differences in stimulus presentation, as our design involved sequentially, not simultaneously presented cross-modal pairings.

We found increased activations to congruent compared to incongruent stimulus pairs in primary auditory cortex. To disentangle whether these activations reflect early integration of context and stimulus (ie feedforward) or instead represent feedback modulations of contextual information on primary sensory areas we examined the effects of context on the carry-over effect on subsequent morphs (or adaptation effect). We show in Fig. 4 that emotional context had no effect on adaptation. We interpret this result as support for late integration of cross-modal information from context and stimulus and suggest that integration occurs further downstream from the sites of adaptation. Our results are consistent with a hierarchical model of vocal emotion perception, in which signals from various sources are initially processed independently and in parallel before being integrated in high-level areas of the temporal lobe.

Although our study focused on emotional context, the observed modulation of auditory regions by visual information may have broader implications for other aspects of voice perception. A prominent model of voice processing proposes parallel systems for extracting identity, speech, and affective cues that interact with corresponding information from faces (Belin et al. 2004). If contextual influences operate via feedback to early auditory areas, as suggested by our findings, similar mechanisms could shape the perception of speaker identity or phonetic detail under ambiguous conditions. For instance, visual context might bias identity judgments when vocal cues are degraded. Future work could examine whether such cross-modal feedback extends beyond emotion to influence linguistic and paralinguistic processing.

Our data provide compelling evidence that situational context significantly influences the perception of vocal emotion, particularly when expressions are ambiguous. Our findings support a hierarchical model of auditory emotion perception in which visual and auditory information are initially processed independently and later integrated in higher-order brain regions. The observed modulation of primary auditory cortex by contextual congruence suggests that feedback mechanisms, rather than early feedforward integration, drive these effects. Our data challenge simplified models of unisensory emotion processing and underscore the importance of cross-modal interactions in shaping emotional perception. By demonstrating that affective congruence enhances neural responses in regions implicated in multisensory integration and cognitive control, our results contribute to a growing body of literature emphasizing the dynamic and context-sensitive nature of emotion perception.
